# The ABBA project (Assess Better Before Access): A retrospective cohort study of neonatal intravascular device outcomes

**DOI:** 10.3389/fped.2022.980725

**Published:** 2022-11-03

**Authors:** Matheus F. P. T. van Rens, Mohammad A. A. Bayoumi, Agnes van de Hoogen, Airene L. V. Francia, Irian J. Cabanillas, Fredericus H. J. van Loon, Timothy R. Spencer

**Affiliations:** ^1^ Neonatal Intensive Care Unit, Women's Wellness and Research Center, Hamad Medical Corporation, Doha, Qatar; ^2^ Department of Neonatology, Wilhelmina Children's Hospital, University Medical Centre of Utrecht, Utrecht, Netherlands; ^3^ Department of Science and Technology in Perioperative Nursing, Fontys University of Applied Sciences, Eindhoven, Netherlands; ^4^ Nursing Department, Global Vascular Access, LLC, Scottsdale, AZ, United States

**Keywords:** neonate, NICU, epicutaneo-caval catheter, peripherally inserted central catheter, peripheral intravenous devices, central venous catheters, intravenous therapy, device-related complications

## Abstract

**Background:**

Venous access devices (VADs) play a vital role within the neonatal intensive care unit. However, there are significant risks associated with the use of VADs, with complications such as infection, thrombosis, device occlusion, and infiltration/extravasation frequently contributing to device-related failures and increasing the risk of significant patient harm or injury. This study aimed to explore the relationships between risk factors and different venous access device complications in the neonatal setting, and then use that evidence to develop an algorithm based on observational data.

**Methods:**

This is a retrospective, single-center cohort study that was conducted in a large 112-bed neonatal intensive care unit in Qatar. We examined venous access device data from January 2016 to December 2018 for all term and preterm neonates. Descriptive statistics were used to summarize the outcomes, which included a mean and its standard deviation or median and an interquartile range for continuous variables regarding normal distribution, and absolute numbers with percentages for discrete variables.

**Results:**

The authors recorded a total of 23,858 VADs inserted during the study period. Of these, 21,313 (89%) were peripheral intravenous catheters, 689 (3%) were extended dwell-peripheral intravenous catheters, 1,335 (6%) were epicutaneo-caval catheters, and 521 (2%) were umbilical venous catheters. In total, 51,179 catheter days were registered, with 2.17 catheter days reported per patient. Peripheral device dwell times were significantly shorter when compared with central venous catheter devices (*P *< 0.001), with mean dwell times of 22 days ± 23 h and 236 days ± 183 h, respectively. After insertion, a complication occurred in 11,177 (51%) of peripheral VADs and 221 (12%) of central VADs. The type of device inserted [*P* < 0.001, hazard ratio (HR) = 0.52, 95% confidence interval (CI): 0.50–0.54], reason/indication for intravenous therapy (*P* < 0.001, HR = 0.85, 95% CI: 0.82–0.87), and the side of insertion of the device (*P* < 0.001, HR = 1.25, 95% CI: 1.24–1.27) had a significant relationship with outcomes.

**Conclusions:**

Four subgroups of VADs were identified (peripheral intravenous catheters, extended dwell-peripheral intravenous devices, epicutaneo-caval catheters, and umbilical venous catheters) with outcome-related differences. Central venous access devices (epicutaneo-caval catheters and umbilical venous catheters) had lower complications compared with peripheral VADs. Proper venous access device selection, early insertion, and early removal approaches remain crucial to preventing venous access device complications. Peripheral intravenous devices should be used carefully and closely watched for early detection of complications.

## Introduction

According to reports, intravenous (IV) access is the most commonly performed invasive procedure in clinical settings worldwide. The support and management of neonatal patient conditions rely tremendously upon the provision of stable venous access for the administration of required and essential fluids, medications, nutrition, and blood products (1). Venous access devices (VADs), either central or peripheral, play a vital role within the neonatal intensive care unit (NICU). However, there are significant risks associated with VAD use, with complications such as infections, phlebitis, thrombosis, device occlusion, and peripheral intravenous infiltration/extravasation (PIVIE) frequently resulting in device failures and increasing the significant risk of patient harm or injury (2). Observational studies have found a total of peripheral IV-related complications including phlebitis, infections, and occlusions as high as 75% (3), and 0%–3% of central catheters in neonates resulted in therapy-related complications and device failure.

Preterm and critically ill neonates are at a higher risk of complications, owing to an immature immune system, an underdeveloped skin barrier, smaller, more fragile blood vessels, and exposure to invasive diagnostic and therapeutic procedures (4–6). Choices in VADs, various catheter insertion techniques, along with care and management strategies, are all complex and multifaceted aspects of care that frequently involve a multidisciplinary team. Evidence-based VAD insertion and maintenance strategies including appropriate skin/insertion site antisepsis and dressings have been developed to reduce the preventable causes of VAD failure and complications; however, evidence in the neonatal population is limited (7, 8). Approaches to VAD selection, insertion, and maintenance in neonates frequently vary among caregivers, with practice often based on traditional practices, anecdotal evidence, or practices recommended for adults and adolescents (9, 10). Undertaking clinical research can help in the identification of evidence-based practices that demonstrate effective methods for reducing preventable causes of patient harm or injury, as well as provide recommendations and guidelines for healthcare practitioners to focus on providing better and safer solutions and outcomes.

Comparing Pettit's benchmark studies (11, 12) with recent literature demonstrates the incidence of neonatal venous access device-related complications has remained persistent over the recent decades, regardless of newer clinical innovations and changes in practices. Infection rates are highly variable for both peripheral and central VADs but have been documented to range between 0.43 and 49 incidents/1,000 catheter days (13–15).

Currently, peripheral intravenous catheters (PIVCs) are most commonly used to provide infusion therapy (16–20). It seems that the presumption of high rates of central line-associated bloodstream infections (CLABSI) plays a significant role in making, often inappropriate, choices of VADs. Comparison studies between CLABSI and peripheral line-associated bloodstream infections (PLABSI) are limited (21–24) and do not indicate any preference for either PIVC or epicutaneo-caval catheter (ECC) (25, 26). Outcomes from a 2015 Cochrane review to determine the effects of infusion of parenteral nutrition *via* percutaneous central venous catheters vs. peripheral cannulae concluded that there was no evidence suggesting that percutaneous central venous catheter use increased the risks of adverse events, particularly invasive infection (27).

Extrinsic modifiable factors may influence successes and failures, highlighting quality clinician training, established competencies, clinical experience, choices regarding device type, patient assessment and insertion techniques, including care and maintenance bundles, device stabilization and securement, and finally dressing protocols (3, 8, 26–28). The application of a transparent dressing plays an important role in reducing the secondary dislodgement of central and peripheral VADs. However, more recently published evidence in the neonatal population has demonstrated the use of cyanoacrylate glue for sutureless securement of epicutaneo-caval catheters might play a significant role in neonates (28). It is well known that each venous access device has its indications, technique of insertion, dwell time, contraindications, and complications. However, evidence from larger-scale studies in these patient populations regarding factors influencing VAD outcomes is still lacking. This study intends to explore the associations between risk factors and different VAD complications in the neonatal setting, and use the evidence to develop an algorithm based on observational data.

## Methods

### Design and setting

In this retrospective, single-center cohort study, anonymized intravenous device data from January 2016 to December 2018 was evaluated. The main study outcome was the occurrence of any venous access device-related complication with the use of the different types of VAD utilized. The study was performed in a large NICU (112 beds) of the Women's Wellness and Research Centre (WWRC) of Hamad Medical Corporation (HMC), Doha, Qatar. The study protocol (MRC-01-19-166) was approved by the above facility's Institutional Review Board (IRB). Because the data source was anonymized, the local IRB deemed that participant consent was not required and determined the study as a “quality chart review.” The project aims to **A**ssess the situation and patients **B**etter **B**efore initiating venous **A**ccess (acronym, ABBA), potentially reducing unwarranted venous device-related complications.

### Patient and public involvement statement

Considering the retrospective design, neither study participants nor parents were involved in the design, conduct or reporting of this study.

### Participants and sample size

All term and preterm neonates who were admitted to the NICU and required intravenous therapy during the NICU stay through a PIVC, ED-PIVC, ECC, or umbilical venous catheters (UVC), were included in the study. Participants were excluded from the study if their data was incomplete or if the data only related to the use of an arterial device, e.g., umbilical or peripheral arterial catheters.

### Procedure

**Figure 1:**
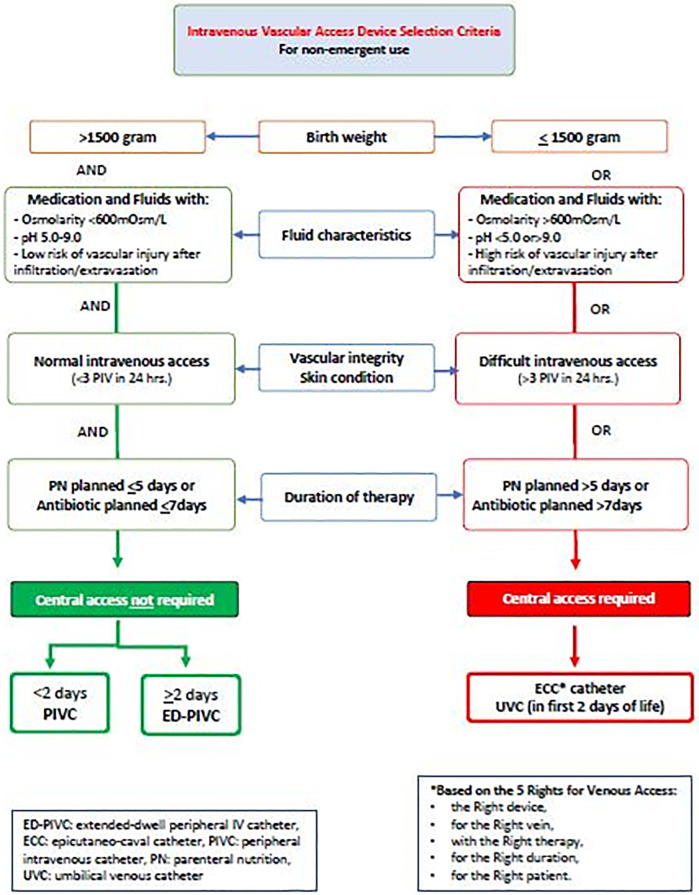
Vascular access device decisional algorithm. ECC, epicutaneo-caval catheter.

In the facility, intravenous cannulation is routinely performed by the neonatal venous access team (NeoVAT). The NeoVAT group is defined as a group of clinicians (nurses and doctors) whose primary role is the assessment of IV access device needs, insertion, oversight of care and use, the management of complications, and collection of venous access device-related data (29, 30). Two notable team delineations are the nurse-led team for peripheral including extended dwell-peripheral IV catheters (ED-PIVC) venous access alone, and a more experienced mixed professional group providing all responsibility for central venous access devices (CVADs). The neonatal ED-PIVC catheter is a short single-lumen catheter that is manufactured in 6 or 8 cm lengths. It is designed to be administered intravenously for up to 29 days. During the patient assessment stage, the team follows a locally developed mnemonic, the “5 Rights for Venous Access,” which stands for the Right device, for the Right vein, with the Right therapy, for the Right duration, for the Right patient, as described in a similar concept by Steere et al. (25) (Figure 1).

Both peripheral and central venous cannulation is performed strictly under the guidance of the local hospital policy and in accordance with the current international standards of practice (27). The selection of suitable veins is performed using the near infrared (NIR) technology for vein visualization, with the saphenous and antecubital veins generally being avoided for peripheral IV cannulation. In this facility's practice, the choice of appropriate venous access device is based on the duration of required therapies; fluid characteristics [pH (5–9) and osmolarity]; patient characteristics, including body weight; and a known history of difficult intravenous access. The design of the venous access device selection flowchart is based on the current infusion standards of practice (27, 31) and local contexts such as product compatibility, hospital purchasing decisions, and practitioner consensus.

### Measurements and data collection

All patient demographics and baseline data included gender, gestational age at birth (in weeks and days), birth weight, and current body weight in grams. Data regarding the procedure for intravenous cannulation were date and time, the number of attempts needed to successful cannulation, cannulation laterality (left, right, or middle), extremity and site/vessel on the extremity (e.g., dorsum of the hand, wrist or forearm, antecubital fossa and upper arm, foot, ankle and lower leg, or knee and upper leg), device type and size (PIVC, ED-PIVC, ECC, UVC), indication for intravenous treatment (intravenous fluids, medication therapy, total parenteral nutrition, blood and/or blood product administration, blood sampling, or procedural, i.e., for power/contrast injection), date and time of device removal, total dwell time in hours (calculated as the removal date and time minus the insertion date and time), reason for removal [therapy completed/discontinuation of therapy warranting (elective) device removal], PIVIE, phlebitis, CLABSI, occlusion, dislodgement and accidental removal, discoloration, and administrative censoring (patient transferred or expired). The data were collected and validated by the authors based from Hamad Medical Corporation.

### Statistical analyses

Descriptive statistics were used to summarize the outcomes, which included a mean and its standard deviation or median and an interquartile range for continuous variables regarding normal distribution, and absolute numbers with percentages for discrete variables. The assumption of normal distribution was proved with Kolmogorov–Smirnov testing. Differences regarding outcomes and measurements were demonstrated by using the *χ*^2^ test, Mann–Whitney *U* test, or unpaired samples *t*-test, as appropriate. A Kaplan–Meier curve was plotted for device dwell time stratified by device type, including its numbers at risk. Hereafter, Cox regression analyses were performed to detect any relationship between independent variables with the outcome of interest. Variables with significance (*P *< 0.05) were included in the multivariable model (main analyses). Using a backward selection process based on the highest Wald score and lowest *P*-value, the smallest set of variables was identified. The hazard ratio (HR) with its 95% confidence interval (CI) was identified in these analyses. Throughout this study, a *P *< 0.05 was denoted to be statistically significant. SPSS (Version 25.0; SPSS Inc., Chicago, IL, United States) was used for statistical analysis.

## Results

During the study period, the authors recorded a total of 23,858 VADs inserted during the study period. In total, 51,179 catheter days were registered, with 2.17 catheter days reported per patient. Of these, 21,313 (89%) were PIVCs, 689 (3%) were ED-PIVC, 1,335 (6%) were ECCs, and 521 (2%) were umbilical venous catheters. Insertion of a VAD was successful in 21,594 patients, resulting in a 90% overall success rate. Individual success rates were observed after the insertion of PIVC (90%), ED-PIVC (92%), ECC (88%), and UVC (96%). In 41% of cases, the VAD was removed after the successful completion of therapy. A complication that may have led to premature VAD removal occurred in 48% of all cases. The study showed an overall complication incidence rate of 260/1,000 catheter days. The most frequently reported failure of therapy was in the PIVC group (411/1,000 catheter days) and the least frequent in the ECC group (10/1,000 catheter days). The risk of therapy failure had four significant variables: the type of VAD, reason or indication for intravenous therapy, dwell time of the VAD (in days), and weight of the patient at the insertion of the VAD. An incidence rate of peripheral line-associated bloodstream infection (PLABSI) was 1.19/1,000 catheter days observed for all peripheral intravenous access devices (PIVC and ED-PIVC), whereas a CLABSI incidence rate of 0.34/1,000 catheter days was observed after CVAD insertion (*P *< 0.001). No other specific complications related to the extended dwell time in CVAD were observed.

A summary of patient characteristics is shown in Table 1. Overall, there was an equal distribution of patients’ gender across the type of devices inserted. The mean gestational age at birth of the total cohort was 34.4 ± 4.8 weeks, and the median age of the patients in days at the moment of insertion of the VAD was 2 (±4) days. A higher number of preterm infants underwent CVAD placement (ECC or UVC), with a mean gestational age at birth of 30.4 ± 4.6 weeks, compared with infants who received a peripheral device (PIVC or ED-PIVC) with a mean gestational age of 34.7 ± 4.6 weeks. Patients who received a peripheral device were slightly older, with a median age of 8 (±5) days, when compared with all infants receiving a CVAD, with a median age of 6 (±4) days. This difference was not statistically significant. Regarding a patient's weight at the time of VAD insertion, those who underwent insertion of a peripheral device had a mean weight of 2,430 ± 928 g and patients who received a CVAD had a mean weight of 1,566 ± 923 g, demonstrating statistical significance. The participants in PIVC and ED-PIVC were, in general, older and had an increased body weight at birth and insertion when compared with other groups; participants in UVC were much younger when compared with other groups, with the oldest participants in PIVC and ECC groups.

**Table 1:** Demographics and baseline parameters.

Variables	PIVC *N* = 21,313	ED-PIVC *N* = 689	ECC *N* = 1,335	UVC *N* = 521
Gender				
Male	12,117 (56.9%)	389 (56.5%)	755 (56.6%)	280 (53.7%)
Female	9,174 (43.0%)	300 (43.5%)	580 (43.4%)	241 (46.3%)
Ambiguous	22 (01%)	0 (0%)	0 (0%)	0 (0%)
Gestational age at birth (weeks)				
<28	2,643 (12.4%)	19 (2.8%)	405 (30.3%)	219 (42.0%)
28–31	1,600 (7.5%)	49 (7.1%)	412 (30.9%)	60 (11.5%)
32–36	6,489 (30.4%)	321 (46.6%)	366 (27.4%)	65 (12.5%)
>36	10,581 (49.6%)	300 (43.5%)	152 (114%)	177 (34.0%)
Weight at birth (g)				
<1,000	2,557 (12.0%)	18 (2.6%)	368 (27.6%)	214 (41.1%)
1,000–1,499	1,752 (8.2%)	25 (3.6%)	528 (39.6%)	58 (11.1%)
1,500–2,500	7,305 (34.3%)	372 (54.0%)	309 (23.1%)	83 (15.9%)
>2,500	9,699 (45.5%)	274 (39.8%)	130 (9.7%)	166 (31.9%)
Age at insertion (weeks)				
<28	725 (3.4%)	0 (0%)	298 (22.3%)	218 (41.6%)
28–31	1,546 (7.3%)	21 (3%)	399 (29.8%)	60 (11.5%)
32–36	7,531 (35.3%)	338 (49.1%)	422 (31.6%)	64 (12.3%)
>36	11,467 (53.8%)	330 (47.9%)	216 (16.2%)	179 (34.4%)
Weight at insertion (g)				
<1,000	1,161 (5.4%)	3 (0.4%	354 (26.5%)	220 (42.2%)
1,000–1,499	2,251 (10.6%)	38 (5.5%)	569 (42.6%)	57 (10.9%)
1,500–2,500	7,983 (37.5%)	374 (54.3%)	257 (19.3%)	82 (15.7%)
>2,500	9,918 (46.5%)	274 (39.8%)	155 (11.6%)	162 (31.1%)

UVC, umbilical venous catheter; PIVC, peripheral intravenous catheter; ED-PIVC, extended dwell-peripheral intravenous catheter; ECC, epicutaneo-caval catheter.

Data are represented as absolute numbers (percentages). This is a retrospective study design and for some parameters, the data values were incomplete due to the unavailability of the information in the patients’ record files thus all the percentage values were computed using nonmissing values.

The primary reason for VAD insertion was to provide continuous infusion for IV therapies and/or medication administration (91%). In general, a median of one attempt was required to obtain successful venous access. See Table 2.

**Table 2:** Data related to the insertion of the devices.

		PIVC *N* = 21,313	ED-PIVC *N* = 689	ECC *N* = 1,335	UVC *N* = 521
Indication for intravenous therapy	IV therapy/medications	19,495 (91.5%)	689 (100%)	1,147 (85.9%)	343 (65.8%)
	Fluid characteristics	0 (0%)	0 (0%)	65 (4.9%)	89 (17.1%)
	Patient characteristics	0 (0%)	0 (0%)	123 (9.2%)	89 (17.1%)
	Blood extraction	1,500 (7%)	0 (0%)	0 (0%)	0 (0%)
	Procedure^a^	318 (1.5%)	0 (0%)	0 (0%)	0 (0%)
Insertion side	Left	11,816 (55.4%)	434 (63%)	402 (30.1%)	0 (0%)
	Right	9,492 (44.5%)	255 (37%)	933 (69.9%)	0 (0%)
	Midline	5 (0.1%)	0 (0%)	0 (0%)	521 (100%)
Insertion site on the body	Ankle	13 (0.1%)	12 (1.7%)	1,030 (77.2%)	0 (0%)
	Elbow	20 (0.1%)	3 (0.4%)	248 (18.6%)	0 (0%)
	Foot	2,448 (11.5%)	415 (60.2%)	0 (0%)	0 (0%)
	Hand	1,808,384.8%)	58 (8.4%)	0 (0%)	0 (0%)
	Knee	0 (0%)	10 (1.5%)	37 (2.8%)	0 (0%)
	Lower arm	582 (2.7%)	53 (7.7%)	0 (0%)	0 (0%)
	Lower leg	27 (0.1%)	64 (9.3%)	10 (07%)	0 (0%)
	Scalp	5 (<0.1%)	0 (0%)	0 (0%)	0 (0%)
	Upper arm	23 (0.1%)	12 (1.7%)	7 (0.5%)	0 (0%)
	Upper leg	6 (<0.1%)	61 (8.9%)	3 (02%)	0 (0%)
	Wrist	106 (0.5%)	1 (0.1%)	0 (0%)	0 (0%)
	Umbilicus	0 (0%)	0 (0%)	0 (0%)	521 (100%)
Number of attempts to cannulation success		1.4 ± 0.7	1.4 ± 0.7	1.4 ± 0.7	1.0 (0.0)
Successful cannulation	Yes	19,284 (90.5%)	637 (92.5%)	1,174 (87.9%)	499 (95.8%)

UVC, umbilical venous catheter; PIVC, peripheral intravenous catheter; ED-PIVC, extended dwell-peripheral intravenous catheter; ECC, epicutaneo-caval catheter; IV, intravenous.

*Data are represented as absolute numbers (percentages), mean ± standard deviation or median (range), as appropriate. This is a retrospective study design and for some parameters, the data values were incomplete due to the unavailability of the information in the patients’ record files thus all the percentage values were computed using nonmissing values. The indications for each device insertion were based on the institutional evidence-based guideline.*

^a^ *VAD insertion related to a procedure if required in diagnostic imaging like MRI, CT scan, etc.*

Data regarding VAD removal are presented in Table 3. The complication rate was highest in the patient who got a PIVC inserted (411/1,000 catheter days), and the lowest complication rate was registered after the insertion of an ECC (10/1,000 catheter days). After the insertion of a peripheral device, a complication occurred in 11,177 (51%) patients, which occurred only in 221 (12%) patients where a CVAD was inserted. In general, for patients who received a peripheral catheter, the complication rate was significantly higher when compared with infants receiving a CVAD (407/1,000 and 13/1,000 catheter days, respectively). Data from 2,713 (11%) patients were denoted as missing values, due to administrative censoring (17%) or a lack of administration (83%).

**Table 3:** Data related to the removal of the devices.

		PIVC *N* = 21,313	ED-PIVC *N* = 689	ECC *N* = 1,335	UVC *N* = 521
Reason for removal	Therapy success	8,318 (43.1%)	197 (30.9%)	903 (76.9%)	329 (65.9%)
	Therapy failure^a^	10,747 (55.7%)	430 (67.5%)	137 (11.7%)	83 (16.6%)
	PIVIE^b^	5,347 (49.7%)	210 (48.8%)	0 (0%)	0 (0%)
	Leaking	2,263 (21.0%)	52 (12.1%)	0 (0%)	0 (0%)
	Phlebitis	1,711 (15.9%)	138 (32.1%)	7 (5.1%)	0 (0%)
	Discoloration	44 (0.4%)	2 (0.46%)	0 (0%)	0 (0%)
	Suspected sepsis	1 (0.009%)	0 (0%)	31 (21.9%)	3 (3.6%)
	Catheter-related complication	0 (0%)	0 (0%)	63 (45.9%)	64 (77.1%)
	Maintenance-related complication	1,381 (12.85%)	28 (6.5%)	37 (27.0%)	16 (19.2%)
Missing	Administrative censoring	225	10	133	87
	Not administrated	2,023	52	161	22

UVC, umbilical venous catheter; PIVC, peripheral intravenous catheter; ED-PIVC, extended dwell-peripheral intravenous catheter; ECC, epicutaneo-caval catheter.

Data are represented as absolute numbers (percentages) or mean ± standard deviation, as appropriate. This is a retrospective study design and for some parameters, the data values were incomplete due to the unavailability of the information in the patients’ record files thus all the percentage values were computed using nonmissing values.

^a^ Therapy failure = PIVIE, leaking (at the insertion site), phlebitis, discoloration, suspected sepsis, maintenance-related complications, catheter-related complications, maintenance-related complications (=accidental removal and occlusion), catheter-related complications (=leaking due to catheter damage, breakage of the catheter).

^b^ PIVIE = peripheral intravenous infiltration/extravasation.

**Figure 2:**
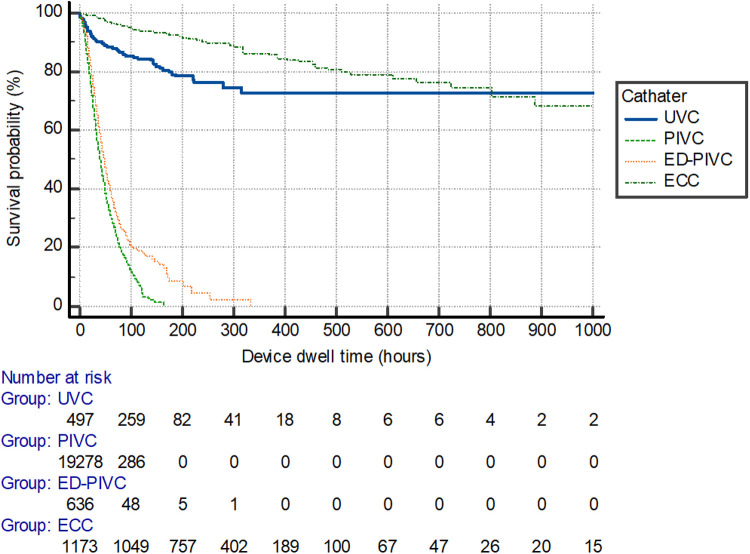
Survival curve per catheter device type for dwell time duration. UVC, umbilical venous catheter; PIVC, peripheral intravenous catheter; ED-PIVC, extended dwell-peripheral intravenous catheter; ECC, epicutaneo-caval catheter.

An overall mean dwell time of 49 ± 78 h was recorded throughout the total cohort of included patients. Peripheral device dwell times were significantly lower when compared with central venous catheter devices (*P *< 0.001), with mean dwell times of 22 days ± 23 h and 236 days ± 183 h. Catheter dwell times differed between the types of devices inserted, as shown in Figure 2 (*P *< 0.001).

Univariable Cox regression analyses identified eight variables demonstrating significant relationships to the outcomes of interest: gestational age at birth, age at device insertion, weight at device insertion, indication for intravenous therapy, type of VAD inserted, chosen site of insertion, side of cannulation, and successful insertion on the first attempt. These variables were all entered for multivariable Cox regression analysis, of which three had a significant relationship with outcomes: the type of device inserted (*P *< 0.001, HR = 0.52, 95% CI: 0.50–0.54), reason/indication for intravenous therapy (*P *< 0.001, HR = 0.85, 95% CI: 0.82–0.87) and side of the insertion of the device (*P *< 0.001, HR = 1.25, 95% CI: 1.24–1.27). Multivariable Cox regression analyses regarding the type of intravenous catheter are represented in Table 4.

**Table 4:** Multivariable Cox regression analyses regarding the type of catheter inserted.

Factor	Beta variable in the outcomes (B)	*P*-value	HR	95% CI
PIVC				
Gestational age at birth	0.05	<0.001	1.05	1.02–1.08
Indication for IV therapy	−0.16	<0.001	0.85	0.83–0.87
Insertion side	−0.04	0.007	0.96	0.93–0.98
Site of insertion on the body	0.15	<0.001	1.16	1.12–1.21
ED-PIVC				
Insertion side	−0.06	0.009	0.95	0.91–0.99
Site of insertion on the body	−2.01	0.042	0.81	0.67–0.98
ECC				
Number of attempts to cannulation success	0.26	0.017	1.30	1.05–1.61
UVC				
Age at insertion	0.03	0.009	1.03	1.01–1.05

UVC, umbilical venous catheter; PIVC, peripheral intravenous catheter; ED-PIVC, extended dwell-peripheral intravenous catheter; ECC, epicutaneo-caval catheter; HR, hazard ratio; CI, confidence interval.

## Discussion

Neonates are an extremely vulnerable and challenging patient population. VADs provide the only gate of all parenterally administered medications and fluids. The incidence of VAD therapy failure may negatively impact clinical therapies and outcomes which are dependent on appropriate, safe, and reliable venous access. Carr et al. (28) determined failure of VADs, resulting in premature device removal, occurred in 48% of participants, with a complication incidence rate of 260/1,000 device days.

While this study provides recent clinical evidence on various risks of therapy failure with intravenous devices in neonates, it has also been reported in previous publications (2, 5, 30). To the best of the authors’ knowledge, a study of this sample size including both peripheral and central catheters, with a focus on device-related failures in neonates, has not been previously published.

To obtain venous access during hospitalization, PIVCs are often sourced as the primary and most inserted devices (5). It is difficult to give an unambiguous clarification for this, although the pattern of complications, and their relative incidences, do correlate.

Results from a small retrospective study performed in a level III NICU stated that the use of an ED-PIVC may be a useful option in providing venous access choices and reducing costs in NICU patients (2, 3). This research compared complications of ECC, ED-PIVC, and PIVC, and when compared with standard PIVCs alone, ED-PIVCs demonstrated advantages in overall device dwell time (ranging from 1 to 29 days), despite that 28% of devices were removed before the completion of therapy. ED-PIVCs also indicated a lower rate of infiltration/extravasation-related episodes when compared with standard PIVCs (1% vs. 4%). The authors concluded at ED-PIVCs offer significant benefits over standard PIVC and moderate financial savings when compared with ECC use and frequent PIVC replacement.

In this study, the use of an ECC reported the least number of therapy failures and longest dwell times. In the study by Chenoweth et al., ECCs had similar success rates compared with peripheral catheters (PIVC and ED-PIVC), when regarding the completion of therapy (*P *= 0.001) (4). Another recent study evaluated the clinical efficacy and safety of ultrasound-guided, subcutaneously tunneled, femorally inserted ECC in the NICU (29). No insertion-related or post-insertion complications were reported, and all patients completed the prescribed therapy with one catheter. However, the device dwell and the device burden on the newborn remain important to mention in this context.

In most NICUs, UVCs are not used for more than 5–7 days, due to increased incidence of late-onset sepsis (LOS) related to UVC longevity. A previous study by Konstantinidi et al. shows the incidence of complications associated with the use of UVCs and ECCs does not significantly differ (30, 32). This current research also confirms these results and shows that ECCs have the benefits of the longest extended dwell times and the lowest incidence of related therapy failures.

There is a significant difference between peripheral and central venous access, hence the knowledge to understand the different causes of failure is essential. Blood flow, vein diameter, type of device materials, and even stabilization and securement (dressing), differ per type of VAD. Facilitating the use of the Right device, for the Right vein, with the Right therapy, for the Right duration, for the Right patient is considered an essential aspect of patient care. However, consistency with international consensus has currently not been achieved.

### Strength and limitations

To the authors’ understanding, this is the first and largest study of this kind to examine the use of VADs and their effect on therapy failures in the neonatal population in this geographical region. All eligible neonates were included, and the sample size was large and representative of the neonatal population requiring VADs. This increased the statistical power of the study's findings, helping to minimize selection bias and increase the generalizability of the findings to similar settings.

Despite these strengths, there are some limitations to this research. This study was conducted in a single center, with a retrospectively collected dataset, and in contrast to randomized studies, this method creates a risk for selection bias. In this study, every infant with a successfully inserted ECC was included to minimize the risk of selection bias. Interrater variability may have affected the results; however, the facility's use of a standardized education program and limiting venous access procedures to members of dedicated venous access team (neoVAT) may have helped reduce data variability. Data outcomes that were not available for neonates (death or were transferred out of the facility) were deemed as “administrative censoring” (in Table 3). Although this population was relatively small, patients lost to follow-up may have different outcomes than those who completed the study. Nonetheless, future research should focus on the introduction of new and clinically beneficial strategies to help improve successful therapy outcomes, no matter the type of VAD.

## Conclusion

In general, many neonatal patients experienced venous access-related complications, resulting in therapy failure. Several variables demonstrated the benefit of the use of an ECC, resulting in the successful completion of prescribed infusion therapies for patients admitted to the NICU.

Four different types of VADs (PIVC, ED-PIVC, ECC, and UVC) were identified, and divided into two groups (central and peripheral), each device with unique characteristics affecting therapy and dwell time in patients admitted to the NICU. Four variables had a significant relationship with therapy failure. Weight (birth and current weight), cannulation site, type of device, and the indication for intravenous treatment all affected the risk for failure. This study demonstrated the highest complication rates were amongst the peripheral VADs when compared with CVADs, including CLABSI. It appears that when a VAD is inserted, it is of utmost importance for the clinician to choose the most appropriate device for the patient and the required therapy. The most frequently observed therapy failure in this neonatal population was with the standard peripheral VAD group. Consequently, the authors concur that peripheral VADs should be used judiciously, and careful thought should be given before their use, particularly when there are alternate means of central access devices that may be utilized more appropriately.

Innovations and clinical advancements in the world of neonatology and VADs with the least chance for therapy failures play an important role in improving patient and therapy-related outcomes. There is clear superiority in using vein visualization technologies like NIR for peripheral venous access and training on simulators and virtual reality for central access devices (32).

The choice of the most appropriate venous device while utilizing the latest technology is an important step for clinicians to place and care for VADs, as well as for patients depending on a reliable venous access device for their required therapy. It is recommended to **A**ssess the patient **B**etter **B**efore inserting the venous **A**ccess device (ABBA) for proper venous access device selection using the VAD algorithm together. Using the “5 Rights for Venous Access” that is, the Right device, for the Right vein, with the Right therapy, for the Right duration, for the Right patient will provide better outcomes and prevent unnecessary catheter-related complications in NICU.

## Data Availability

The datasets generated for this study are available on reasonable request to the corresponding author.
